# Phytochemical Characterization and Screening of Antioxidant, Antimicrobial and Antiproliferative Properties of *Allium × cornutum* Clementi and Two Varieties of *Allium cepa* L. Peel Extracts

**DOI:** 10.3390/plants10050832

**Published:** 2021-04-21

**Authors:** Željana Fredotović, Jasna Puizina, Marija Nazlić, Ana Maravić, Ivica Ljubenkov, Barbara Soldo, Elma Vuko, Danica Bajić

**Affiliations:** 1 Department of Biology, Faculty of Science, University of Split, R. Boškovića 33, 21000 Split, Croatia; puizina@pmfst.hr (J.P.); mnazlic@pmfst.hr (M.N.); amaravic@pmfst.hr (A.M.); elma@pmfst.hr (E.V.); dbajic@pmfst.hr (D.B.); 2 Department of Chemistry, Faculty of Science, University of Split, R. Boškovića 33, 21000 Split, Croatia; iljubenk@pmfst.hr (I.L.); barbara@pmfst.hr (B.S.)

**Keywords:** *Allium × cornutum* Clementi ex Visiani, 1842, *Allium cepa* L. red and yellow variety, flavonols, anthocyanins, antioxidant properties, antimicrobial and antiproliferative activity

## Abstract

Onions are one of the most widely grown vegetable crops. As production increases, so does the generation of waste from various parts of the onion, raising the need for efficient ecological disposal and use of such waste products. However, onion waste products are a rich source of antioxidants with a range of biological properties, therefore, they could potentially be used in food and pharmaceutical industries. In the present study, we identified the main flavonols and anthocyanins in peel extracts of *Allium × cornutum* Clement ex Visiani, 1842, and two varieties of *Allium cepa* L. and tested their antioxidant, antimicrobial and antiproliferative properties. Quercetin 3,4′-diglucolside, quercetin 4′-monoglucoside and quercetin are the most abundant flavonols in all onion extracts detected by high-performance liquid chromatography (HPLC) method. The composition of anthocyanins varied in all extracts. 2,2′-diphenyl-1-picrylhydrazyl (DPPH) and oxygen radical absorbance capacity (ORAC) assays showed that the triploid onion *A. × cornutum* had the highest antioxidant power. Evaluation of antimicrobial activity by broth microdilution assay also showed that *A. × cornutum* had higher antimicrobial activity compared to the red and yellow onion varieties. Comparable antiproliferative activity was confirmed for all onion extracts tested on three cancer cell lines: Hela (cervical cancer cell line), HCT116 (human colon cancer cell line) and U2OS (human osteosarcoma cell line). The most abundant onion flavonols (quercetin 3,4′-diglucoside and quercetin 4′-monoglucoside) showed weaker antimicrobial as well as antiproliferative properties compared to the extracts, leading to the conclusion that other phytochemicals besides flavonols contribute to the biological activity of onion peel extracts. The results demonstrate the antioxidant and antimicrobial properties of onion peels, which have promising potential as cancer cell proliferation inhibitors.

## 1. Introduction

*Allium* species, especially *A. cepa* (common onion), are one of the most important crops worldwide. *Allium × cornutum* (2n = 3x = 24) is an established small garden crop widely distributed in southeastern Asia and Europe. Combined molecular, phylogenetic and cytogenetic analysis provided evidence for its unique genetic structure and triploid genomic origin with three putative parent species of section Cepa: *A. cepa*, *A. roylei* and the wild Asian species *Allium pskemense* B. Fedtsh [1]. These onions are edible species and have been used in human nutrition for years. Both production (about 105 billion pounds per year) and consumption have increased by over 70% in the last twenty years. Over 500,000 tons of onion waste is generated annually in the European Union, mainly in Spain and the Netherlands. Onion peels discarded during peeling become a major environmental (rapid growth of phytopathogens) and economic problem (40 €/ton disposal cost) [2]. Recently, scientists have attempted to explore the potential benefits of onion waste. It has already been shown that *Allium* vegetables are a source of important phytochemicals, mainly sulfur compounds and polyphenols [3,4,5,6]. However, it should be mentioned that the amount of these compounds in onion waste is significantly higher than in the edible parts of onions [7]. Until recently, flavonoids were thought to be absorbed by passive diffusion after glycosylated flavonoids were converted to aglycones following consumption of onion meal [8]. However, studies have shown that quercetin glycosides are better absorbed than quercetin aglycones in humans [9,10,11,12]. After consumption, flavonol conjugates are absorbed mainly in the stomach and/or small intestine. Flavonoids that are not absorbed in the stomach or small intestine enter the colon, where they undergo a process of deglycosylation and deconjugation and are broken down by the action of colon bacteria. Even after degradation, they influence the redox balance by stimulating the activity of antioxidant enzymes and detoxifying enzymes or leading to the formation of compounds involved in the maintenance of homeostasis [13]. Phenolic compounds show significant antioxidant, anti-inflammatory, antiallergic, antimicrobial and anticancer activity [14,15,16,17,18,19,20,21,22]. These properties are closely related to the structure of the most abundant phenolics, the quercetin and its conjugates [5,15,23]. It has been shown that the antioxidant activity of garlic and various onion varieties is significantly correlated with high total phenolic content (TPC) [24]. A previous study confirmed that the addition of onion components to the high cholesterol diet of rats reduced oxidative stress by stimulating the activity of the antioxidant system [25]. It has been reported that consumption of onions—a food rich in flavonoids—may increase the resistance of the DNA of human lymphocytes to DNA strand breaks caused by oxidative stress [26]. The strong antioxidant activity makes them one of the best antioxidants and radical scavengers that could be used not only for human nutrition, but also as a source of natural antioxidants and for pharmaceutical purposes. Several studies have shown that organosulfur compounds alone are responsible for the antimicrobial activity of onions. They successfully inhibited the growth of gram-positive bacteria belonging to the genera *Bacillus*, *Micrococcus*, *Staphylococcus*, *Streptococcus*, as well as some gram-negative bacteria such as *Escherichia coli* and *Salmonella enteritidis* [27,28]. In addition to sulfur compounds, flavonols, especially the oxidation products of quercetin present in onions, strongly inhibited the growth of *Helicobacter pylori* and MRSA (multidrug-resistant *Staphylococcus aureus*) [29]. The antiproliferative activity of *Allium* species has been demonstrated in many in vitro and in vivo studies as well as in epidemiological studies [30,31,32]. This effect is related++ with both phenolic and organosulfur compounds. Quercetin glycosides (q 3,4′-diglucoside and q 4′-monoglucoside) isolated from four onions, *A. chinese* (Chinese onion), *A. sativum* (garlic), *A. cepa* L. (common onion) and *A. fistulosum* L. (Welsh onion), effectively inhibited the growth of HepG2, PC-3 and HT -29 cancer cells [33]. Therefore, the combination of quercetin glucosides might be crucial for the antiproliferative activity of onion extracts. A study by Chang et al. showed that these compounds can act as activators of apoptosis in cancer cells [34]. Flavonoids can reduce the activity of enzymes such as ornithine decarboxylase and degrade polyamines, preventing DNA synthesis and cancer cell proliferation [35,36,37]. Onion volatile sulfur compounds, mainly diallyl disulfides and diallyl trisulfides, play an important role in the antiproliferative activity of onions. They have been shown to reduce the growth of colon, lung and skin cancer cells and promote apoptosis in HL60, HCT-15 and neuroblastoma cells by increasing the ROS level and inducing cell death [38,39,40,41,42]. Epidemiological studies have shown the link between the consumption of *Allium* vegetables and a lower risk of developing lung, gastrointestinal, breast and brain cancer [16,43,44,45,46,47]. Most of these studies have been performed with edible onion bulbs, but very few studies have been conducted to evaluate the health effects of onion waste [27,28,32,48,49,50]. Considering the above-mentioned properties and beneficial effects on health, and the fact that the peel extracts of triploid hybrid onion *A. × cornutum* have never been chemically characterized or tested for their biological activity, the aim of our study is (a) to identify the compounds present in the methanolic extracts of the outer scales of two onions traditionally grown in Dalmatia (*A. × cornutum* and *A. cepa*) and (b) to investigate their antioxidant, antimicrobial and antiproliferative activities.

## 2. Results and Discussion

### 2.1. Flavonol and Anthocyanin Determination by HPLC

HPLC analysis of extracts from peel waste of triploid onion (*A. × cornutum* Clementi ex Visiani) and two varieties of diploid onion (*A. cepa* L.) revealed the presence of flavonols and anthocyanins in significantly higher amounts than previously identified in onion bulb extracts [51,52]. Five different flavonols were detected in all extracts: quercetin 3,4′-diglucoside (1), quercetin 4′-monoglucoside (2), quercetin aglycone (3), isorhamnetin (4) and kaempferol (5), as shown in the chromatograms (Figure S1). The two major flavonols identified were quercetin-3,4′-diglucoside and quercetin-4′-monoglucoside, the concentration of which was at least twice as high in the triploid onion *A. × cornutum* as in the other two diploid onion cultivars (Table 1). Compared to the results of the study with onion bulb extracts [5], the concentration of all flavonols is significantly higher in the peel extracts, almost three times higher in the peel extract of *A. × cornutum* and twice as high in the peel of the red onion variety than in its bulb extracts. Similar results, confirming the concentration of each flavonol glucoside ((1), (2) and (3)) have been reported previously [53,54,55,56]. Quercetin and its derivatives are important dominant constituents of onions. They have the role of antioxidants and free radical scavenging properties along with the ability to protect against various diseases. Considering the increased amount of these important antioxidants in onion peels as compared to onion bulbs, it would be extremely important to utilize the potential of onion peels that remain unused after processing.

The anthocyanins identified in onion waste extracts are shown in Table 1. Five anthocyanins were detected in the red and yellow varieties of *A. cepa*, while four anthocyanins were present in the extract of *A. × cornutum*. Interestingly, there are differences in anthocyanin composition in all the extracts tested. Delphinidin 3′-glucoside acetate was found only in *A. × cornutum*, as previously reported by Gennaro et al. [57] and Fredotović et al. [5]. Peonidin-3′-glucoside acetate and petunidin-3′-glucoside acetate were present only in the red variety of *A. cepa*, while peonidin and petunidin-3′-glucoside were identified only in the yellow onion variety. The most abundant anthocyanin in all extracts was cyanidin-3-glucoside acetate and cyanidin-3-glucoside, the concentration of which was by far the highest in the yellow onion (7.85 ± 0.11 mg/100 g dry weight). Interestingly, these anthocyanins were not found in measurable amounts in the extracts of onion bulbs reported by Fredotović et al. [5]. Malvidin-3′-glucoside was also identified in all onions. The differences in anthocyanin composition and concentration are most likely due to genetic variations [58].

### 2.2. Antioxidant Activity of Onion Peel Extracts

The antioxidant activity of onion waste extracts was determined using 2,2′-diphenyl-1-picrylhydrazyl (DPPH) and oxygen radical absorbance capacity (ORAC) methods. Several researchers have shown a close correlation between antioxidant capacity and phenolic content of extracts from various natural sources [56,59,60]. Since onion waste extracts are rich in phenolic compounds (Table 1), which are known for their ability to act as electron or hydrogen donors, they were expected to have a significant antioxidant capacity. Both Nile A. et al. [53] and Nile S.H. et al. [54] reported the antioxidant activity of the major flavonols (quercetin 3,4′-diglucoside, quercetin 4′-monoglucoside and quercetin aglycone) present in methanolic onion waste extracts. Pure quercetin showed the highest antioxidant capacity followed by quercetin diglucoside and then quercetin monoglucoside. The results showed that the antioxidant activity of onion extracts was correlated with phenolic content. However, the influence of organosulfur compounds on the antioxidant power of onions cannot be excluded [59,61,62]. As shown in Table 2, the extract of *A. × cornutum* showed significantly higher antioxidant activity than the red and yellow onion varieties in both DPPH and ORAC assays at a concentration of 100 µg/mL, which can be explained by the higher concentration of phenolic compounds. Despite the fact that the yellow onion variety has the lowest concentration of all identified flavonols, it dominates in the amount of the anthocyanin cyanidin-3-glucoside, which has shown strong antioxidant activity due to its hydroxyl group rich structure [58,63]. This could explain the better DPPH and ORAC value of the yellow onion variety compared to the red variety. Škerget et al. analyzed DPPH activity for 35% acetone and 60% ethanolic extracts of yellow onion peel. They reported values of 46.95 ± 1.02% and 47.14 ± 1.85%, respectively [28]. The antioxidant activity for yellow onion peel extract is in agreement with other results reported by Lee et al. [64]. The antioxidant DPPH activity for the ethanolic extract of yellow onion peel was 72.25 ± 2.74% and for the water extract was 49.68 ± 1.55%. Nile et al. reported the following DPPH activity values for aqueous methanol, aqueous ethanol and ethyl acetate extracts of solid onion waste: 74.3 ± 1.1%, 60.5 ± 1.4% and 54.6 ± 1.8%, respectively [53]. Compared with the results of our previous study on onion flesh extracts [5], the present results showed that the antioxidant power increased from the inner to the outer parts of the onion. These results are consistent with those of Benítez et al. [55] and Nuutila et al. [65].

### 2.3. Antimicrobial Activity of Extracts and Two Major Flavonols (Quercetin 3,4′-Diglucoside and Quercetin 4′-Monoglucoside)

Many studies have highlighted the influence of organosulfur compounds in onions as important inhibitors of pathogenic bacteria growth [28,29]. However, the antimicrobial properties of phenolic compounds have also been confirmed in some studies [29,66,67,68,69,70,71]. The results of antimicrobial activity of onion waste extracts and quercetin conjugates are presented in Table 3 and Table 4. All the extracts showed better inhibitory activity on gram-positive bacteria as compared to gram-negative. Similar results were obtained by Corzo-Martínez et al. [27] and Škerget et al. [28]. The extracts of *A. × cornutum* showed higher activity on all tested bacteria, yeasts and molds compared to yellow and red onion varieties. The yellow variety of *A. cepa* was more effective than the red variety. Both *A. × cornutum* and the yellow variety of *A. cepa* strongly inhibited the growth of the two *Staphylococcus aureus* strains (Clinical/MRSA and ATCC 29213) with MIC (minimum inhibitory concentration) and MBC (minimum bactericidal concentration) values: 7 µg/mL and 125 µg/mL, respectively. These results are in agreement with previously reported results for onion bulb and waste extracts [27,68,70]. *Streptococcus pyogenes* (MIC 31.25 µg/mL) and *Listeria monocytogenes* (MIC 15.6 µg/mL) were more sensitive to *A. × cornutum* extract than *Bacillus cereus* (MIC 125 µg/mL). *Enterococcus faecalis* was the least sensitive with an MIC value of 250 µg/mL. Both *A. cepa* varieties showed weak or no inhibition of the growth of these bacteria. Gram-negative bacteria were significantly more resistant to all extracts. Again, *A. × cornutum* showed better inhibitory activity than the other two onions with an extremely high MIC value of 500 µg/mL. A similar effect was observed by Santas et al. [68]. They showed that the ethyl acetate subfraction of crude onion extract inhibited the growth of *Bacillus cereus*, *Staphylococcus aureus*, *Micrococcus luteus* and *Listeria monocytogenes*, but had no effect on gram-negative bacteria *Eschericia coli and Pseudomonas aeruginosa*. No antifungal activity of *A. × cornutum* and *A. cepa* waste extracts against *Candida albicans* was observed, which is in agreement with previously published data [29]. Food-poisoning mold, *Aspergillus niger* was not affected by either the red or yellow *A. cepa* variety. *A. × cornutum* showed low activity against this fungus with an MIC value of 500 µg/mL. Škerget et al. [28] investigated the antifungal activity of onion bulb and peel extracts. The authors indicated that the bulb extract showed lower efficacy in inhibiting the growth of *A. niger* than the onion peel extract. They explained this by the higher concentration of bioactive compounds in the onion peel.

As mentioned in Section 2.1., onion peel extracts are rich in glucosidic forms of phenols, mainly quercetin 3,4′-diglucoside and quercetin 4′-monoglucoside, which we also tested for antimicrobial activity. To our knowledge, there are no data on the antimicrobial activity of dominant quercetin conjugates of onion waste extracts. There are few studies on flavonoid glycosides, mainly flavonol 3-O-glycosides, which showed strong antibacterial activity against gram-positive bacteria and low activity against gram-negative bacteria [70].

Quercetin 3,4′-diglucoside and quercetin 4′-monoglucoside showed similar inhibitory effects on almost all microbes tested, except *Enterococcus faecalis* and *Aspergillus niger*, which were slightly better affected by the monoglucoside form of quercetin (Table 4). The antimicrobial activity of the pure quercetin conjugates was generally lower than that of the *A. × cornutum* extract and the yellow variety of the *A. cepa* extract, except for the Gram-negative bacteria *E. coli* and *K. pneumoniae* and the food-poisoning mold *Aspergillus niger*. Several studies have shown that the glycoside forms of phenols have weaker activity than phenol aglycones because they lack a hydroxyl group on the C-3 atom, which has been shown to be necessary for antimicrobial activity [28,29,70]. The results of the present study suggest that there are other biologically active components in onion extracts besides flavonols that are responsible for antimicrobial activity. Further research is needed to determine the synergistic effect of the bioactive components of onion extracts.

### 2.4. Antiprioliferative Activity of Onion Extracts and Two Major Flavonols (Quercetin 3,4′-Diglucoside and Quercetin 4′-Monoglucoside) on HeLa, HCT116 and U2OS Cancer Cell Lines

**Figure 1:**
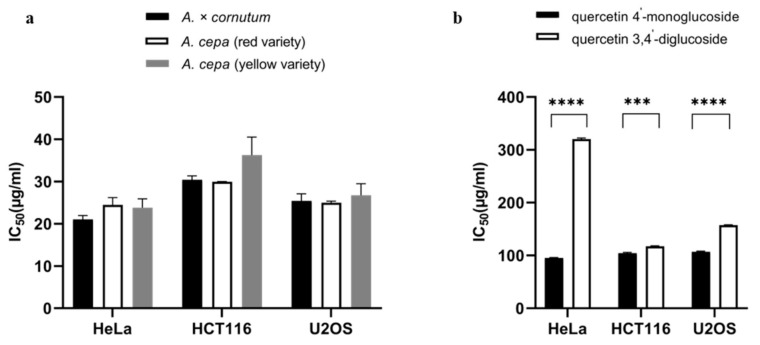
Antiproliferative activity of onion waste extracts (**a**) and quercetin conjugates (**b**) determined by MTS-based cell proliferation assay. The results are expressed as mean values of three independent experiments ± SD (standard deviation). Statistically significant differences are marked with *** *p* < 0.001 and **** *p* < 0.0001 (Data S3 and S4).

The antiproliferative activity of the *A. × cornutum* and two varieties of *A. cepa* extracts as well as the flavonols were tested against three cancer cell lines using an MTS cell proliferation assay. Cells were treated with various concentrations of onion extracts and incubated for 48 h. Subsequently, the half maximal inhibitory concentration (IC_50_) value was determined. In contrast to the results of antioxidant and antimicrobial activity, in the case of antiproliferative activity, the red variety of *A. cepa* proved to be better than the yellow variety and *A. × cornutum* in inhibiting the proliferation of two cancer cell lines, HCT116 and U2OS (Figure 1a). These results are in correlation with the previously reported study conducted with *A. × cornutum* and *A. cepa* bulb extracts [6].

*A. × cornutum* and yellow variety of *A. cepa* showed similar and slightly better inhibitory activity on HeLa cells than the red variety of *A. cepa*. Similar results were obtained for other *Allium* extracts on different cancer cell lines. Murayyan et al. [72] showed that five Ontario grown onion varieties exhibited potent antiproliferative activity against human adenocarcinoma cells (Caco-2). Kim et al. [48] confirmed the cytotoxic effect of ethanol extract of onion peel on human colon carcinoma cells (HT-29). Similar results were obtained by Xu et al. [73] who indicated that the watery extract of *A. ursinum* inhibited proliferation and induced apoptosis in human AGS gastric cancer cells. Nile et al. [53] showed that not only the onion solid waste (OSW) extract, but also each extracted flavonol glucoside from the OSW extract was cytotoxic to human renal carcinoma (ACHN), human pancreatic carcinoma (Panc1), human non-small lung carcinoma (Calu 1), human non-cell lung carcinoma (H460) and human colon cancer (HCT116).

The good antiproliferative activity of onions is certainly due in part to the quercetin glycosides, which have been identified as the major bioactive components of the extracts. However, it is evident that there are other biologically active molecules that also contribute to the antiproliferative activity. The present results (Figure 1b) show that the quercetin glycosides have moderate activity on the tested cancer cells compared to the extracts. Quercetin 4′-monoglucoside showed a lower IC_50_ value on all cells, indicating that it has a significantly better antiproliferative activity than the second most important quercetin glycoside present in the onion peel extracts—quercetin 3,4′-diglucoside. Previous studies have shown that the combination of quercetin glycosides may modulate the antiproliferative activity of onion extracts on various cancer cells [33,74,75], They also suggested that quercetin glycosides can activate apoptosis in various cancer cell lines. Their action could also be related with their ability to inhibit prooxidant processes or the enzyme xanthine oxidase, and in this way prevent cancer proliferation [75,76].

The biological activity of flavonols from *Allium* plants depends largely on their bioavailability. The bioavailability of quercetin and its glucoside compounds is perhaps one of the most studied, precisely because they contribute to the major intake in humans. Despite many studies, their bioavailability in the human body has not been fully elucidated. Many factors influence the site and manner in which flavonols are absorbed, and it mainly depends on their chemical structure [77]. We performed in vitro digestion of onion extracts to determine the stability of flavonols and the effect on the antiproliferative activity.

**Figure 2:**
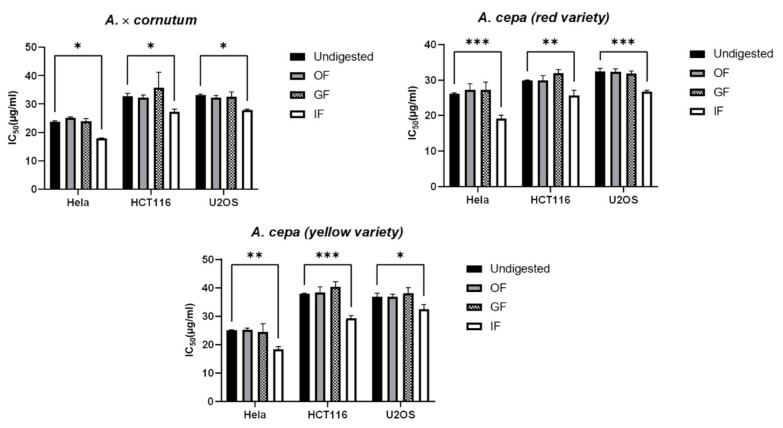
Antiproliferative activity of digested onion waste extracts determined by MTS-based cell proliferation assay. The results are expressed as mean values of three independent experiments ± SD (standard deviation). Statistically significant differences are marked with * p < 0.05, ** *p* < 0.01 and *** *p* < 0.001 (Data S5–S7). Undigested—control extract, OF—oral phase, GF—gastric phase and IF—intestinal phase.

The results showed that the extracts remained quite stable after oral and gastric digestion, as we found no significant differences in antiproliferative activity compared to undigested extracts. This is in correlation with previous studies which confirmed that most polyphenols are relatively stable under gastric digestion [78,79]. In vitro small intestinal digestion increased the antiproliferative activity of the extracts on all cancer cells tested compared to the undigested (control) extract (Figure 2). There are numerous reports on the increased bioactivity of plant extracts after in vitro stimulated digestion. The authors generally reported increased antioxidant and antiproliferative activity [78,80,81,82]. In addition, they state that during digestion, compounds are transformed into those with a different chemical structure and pharmacological properties. We assume that quercetin glycosides might be transformed into different structural forms such as quercetin aglycone. Murota and Terao [83] reported that quercetin glucosides are better absorbed in the stomach than quercetin aglycone, due to their water solubility. The quercetin aglycone is relatively lipophilic, which is why it can pass the intestinal barrier more easily by passive transport, unlike the glycosides which are hydrophilic due to their structure rich in sugar moieties. An increase in quercetin amount after in vitro digestion was reported by Hur et al. [78]. They showed that the amount of quercetin in onion extract as well as its antioxidant activity increased significantly more after in vitro digestion in the small intestine than after digestion in the mouth and stomach. It seems that the reason could be the difference in pH or enzymes between stomach and small intestine. Other factors, such as temperature, oxygen or enzymes, may also have an influence on the phenolic composition [80,81,82,83,84].

In conclusion, this study provided evidence for the possibility of using onion peel extracts as cytotoxic agents that could potentially reduce the risk of cancer development. It is necessary to conduct further in vitro and in vivo studies, both with the extracts and with individual compounds of the extracts, in addition to further bioavailability studies, in order to reveal the mechanism of action and to open up the possibility of their use as chemotherapeutic agents.

## 3. Materials and Methods

### 3.1. Collection of Plant Material and Preparation of Onion Waste Extracts

*Allium × cornutum* Clementi ex Visiani (Ljutika) was obtained from local gardens along the Dalmatian coast and *Allium cepa* L. (Red Baron and Holandska rumena (Stuttgarter reisen) varieties) was purchased at the local market with domestic products. Outer layers of onion bulbs were separated from edible parts, washed and air-dried overnight. Then the peels were freeze-dried and homogenized with 70% methanol water. The samples were extracted three times for 30 min with magnetic stirring at room temperature (ca. 20 °C) and the homogenate was centrifuged at 3000 rpm for 15 min. The supernatants were pooled and the solvent was removed using a rotary evaporator (approx. 50 °C). The procedure was repeated until a constant sample weight was obtained. The onion peel extracts were dissolved in 10% DMSO and filtered through a 0.45 µm nylon filter before use.

### 3.2. Phytochemical Characterization of Extracts

Flavonols were measured by high-performance liquid chromatography (HPLC) using the Perkin Elmer HPLC system (Waltham, MA, USA), which consists of a binary pump Series 200, an autosampler, Peltier column oven Series 200, UV-VIS detector Series 200, and an UltraAqueous C18 column (250 × 4.6 mm, Resek, Bellefonte, PA, USA). The TotalChrom Workstation software (version 6.2.1, Perkin Elmer, Waltham, MA, USA) was used to process the chromatographic data. Anthocyanins were analyzed using a Varian HPLC system (Varian, Inc., Harbour City, CA, USA) consisting of a Star 9010 pump, a Rheodyne 7125 syringe-charge sample injector, a 500- LC module for a column oven, a ProStar 330 photodiode array detector, and a Star Chromatography workstation, version 5. Separation was performed using a Kinetex C18 core-shell column (150 × 4.6 mm) filled with 5 µm particles and equipped with a SecurityGuard ULTRA Cartridge UHPLC C18 for 4.6 mm ID column (Phenomenex, Torrance, CA, USA), both thermostated to 35 °C. HPLC analysis was performed according to Fredotović et al. (2017) [5]. Identification of flavonols and anthocyanins was based on their retention times compared to the standards. Quantification of each compound was determined using the calibration curves of the external standard. Quercetin, quercetin 4′-monoglucoside Sigma-Aldrich (St. Louis, MO, USA), and quercetin 3,4′-diglucoside (Polyphenols AS, Sandnes, Norway) prepared in methanol were used as standards for the identification of flavonols, and malvidin 3-*O*-glucoside chloride (Extrasynthese, Genay, France) for anthocyanins. The amount of each compound was expressed as mg/100 g dry weight. Retention time (RT), limit of detection (LOD), limit of quantification (LOQ), and coefficient of correlation (R2) for flavonols and anthocyanins are presented in Supplementary material (Data S8).

### 3.3. Antioxidant Activity of Onion Peel Extracts

#### 3.3.1. Oxygen Radical Absorbance Capacity Assay (ORAC)

The assay was performed in a Perkin–Elmer LS55 spectrofluorimeter, using 96-well white polystyrene microtiter plates (Porvair Sciences, Leatherhead, UK) according to a method described by Fredotović et al. [5]. Each reaction contained 180 µL of fluorescein (1 µM), 70 µL of 2,2′-Azobis(2-methyl-propionamidine) dihydrochloride (AAPH, Acros Organics) (300 mM), and 30 µL of plant extract or reference standard Trolox (6.25–50 µM) (Sigma–Aldrich). All experimental solutions were prepared in a phosphate buffer (0.075 mM, pH 7.0). The extract was prepared in methanol (1 mg/mL) and further diluted in a phosphate buffer until the concentration was 15.6 µg/mL. These solutions were further diluted 40× and 80× with the phosphate buffer for the experiments. Measurements were performed in triplicate. ORAC values were expressed as µmol of Trolox equivalents (TE) per mL of extract (for concentration 100 µg/mL).

#### 3.3.2. Measurement of DPPH Radical Scavenging Activity

The antioxidant capacity of the extracts was determined by the DPPH method previously used by Nazlić et al. [85]. This method uses 96-well microtiter plates for the reduction reaction of alcoholic DPPH (2,2-diphenyl-1-picrylhydrazyl) solution (Sigma–Aldrich) in the presence of a hydrogen-donating antioxidant. Plant extracts as described in the ORAC method were used (methanolic onion peel extracts). After the final step of adding 100 µL of a methanolic solution of DPPH (200 µM) to each well, the reaction starts and the initial absorbance was immediately measured at 517 nm, using MeOH as a blank. After 30 and 60 min incubation, the absorbance was measured again and the percentage of DPPH inhibition was calculated according to the following formula of Yen and Duh [86]:

% inhibition = ((A_C_(0) − A_A_(*t*))/A_C_(0)) × 100,

where A_C_(0) is the absorbance of the control at *t* = 0 min, and A_A_(*t*) is the absorbance of the antioxidant at *t* = 1 h. All measurements were performed in triplicate.

### 3.4. Antimicrobial Activity of Onion Extracts and Quercetin Conjugates

#### 3.4.1. Microbial Strains

To assess antimicrobial activity, extracts of *A. × cornutum* and *A. cepa* peels, as well as quercetin 3,4′-diglucoside and quercetin 4′-monoglucoside were tested against eleven strains of human opportunistic pathogens and food-spoilage microorganisms. The antimicrobial tests included Gram-negative *Escherichia coli* ATCC 25922 and *Klebsiella pneumoniae* ATCC 13883, and five Gram-positive species: *Staphylococcus aureus* (ATCC 29213 and a methicillin-resistant *S. aureus* clinical strain MRSA-1), *Listeria monocytogenes* ATCC 19111, *Streptococcus pyogenes* ATCC 19615, *Enterococcus faecalis* ATCC 29212 and food-borne *Bacillus cereus* isolate. The antifungal efficacy was evaluated against the opportunistic yeast *Candida albicans* ATCC 90029 and food-spoilage mold *Aspergillus niger*.

#### 3.4.2. Broth Microdilution Assays

Broth microdilution assays were performed according to the European Committee on Antimicrobial Susceptibility Testing (EUCAST) protocols for bacteria (EUCAST 2020a) [87] and fungi (EUCAST 2020b, 2020c) [88,89]. Sabouraud dextrose broth (SDB; Biolife Italiana, Milano, Italy) was used for fungal growth.

All solutions were prepared in 10% DMSO. Extracts of *A. × cornutum* and *A. cepa* peels (10 mg/mL) were tested in the concentration range of 2000 to 1.95 µg/mL. Quercetin 4′-monoglucoside (1.5 mg/mL) was tested at concentrations ranging from 300 to 0.3 µg/mL, while in the case of quercetin 3,4′-diglucoside (1 mg/mL) concentration ranged from 200 to 1.9 µg/mL. The assays were performed in 96-well polystyrene microtiter plates as previously described [90]. Briefly, exponentially grown bacterial cultures in Mueller-Hinton broth (MHB; Biolife) were spectrophotometrically adjusted to 10^6^ CFU/mL, added to serial two-fold dilutions of extracts and compounds in a final volume of 100 µl per well, and incubated for 18 h at 37 °C. In the case of fungi, an inoculum of approximately 2.5 × 105 CFU/mL of spores/conidia was added to the wells and incubated at 35 °C for 24 h (*C. albicans*) and 48 h (mold). The minimal inhibitory concentration (MIC) was defined as the lowest concentration that showed no visible bacterial growth (turbidity) in the wells. To determine the minimum bactericidal concentration (MBC), aliquots were taken from the wells corresponding to MIC, 2xMIC and 4xMIC and plated on MHA plates. After incubation at 37 °C for 18 h, the MBC value was determined as the lowest concentration causing ~99.9% killing of the starting inoculum.

For fungi, aliquots from the wells were plated on SDA and incubated for 24 and 48 h at 35 °C. After counting colonies, MIC_50_ and MIC_90_ endpoints were recorded as the lowest concentrations that inhibited 50% and 90% of the fungal growth compared to the control. All tests were performed in triplicate.

### 3.5. Antiproliferative Activity

#### 3.5.1. Antiproliferative Activity of Onion Extracts and Quercetin Conjugates

The antiproliferative assay was performed in the same way and with the same cancer cells (cervical cancer cell line—HeLa, human colon cancer cell line—HCT116 and human osteosarcoma cell line—U2OS) as described by Fredotović et al. [6], to compare the results obtained for onion bulb extracts with those obtained for onion waste extracts. The cells were a gift from the laboratory of Janoš Terzić at the School of Medicine, University of Split. Cells were grown in a CO_2_ incubator at 37 °C and 5% CO_2_ until they reached 80% confluency. They were then seeded in 96-well plates (approximately 5000 cells per well) and treated with serial dilutions of the extracts. The plates were left in the incubator for 48 h. Then, 20 µL of MTS tetrazolium reagent (Promega) was added to each well, the 96-well plate was incubated at 37 °C and 5% CO_2_ for another 3 h, and then the absorbance was measured on a microplate reader (Bio-Tek, EL808). The IC_50_ value was calculated as the mean of three independent experiments using GraFit 6 data analysis software (Erithacus, East Grinstead, UK).

#### 3.5.2. Antiproliferative Activity of Onion Extracts after In Vitro Digestion

The antiproliferation assay was performed in the same manner as described in Section 3.5.1., except that this time the cells were treated with digested peel extracts of onions. In vitro digestion was performed as described in previous studies [82,91]. Oral phase: 5 g of the onion extract was mixed with 3.5 mL of SSF (stimulated salivary fluid) and 0.5 mL of α-amylase solution (1500 U/mL, Sigma-Aldrich, Saint Louis, MO, USA), followed by the addition of 25 mL of 0.3 M CaCl_2_ and 975 mL of water and shaken vigorously. Gastric phase: 10 mL of the previously treated sample was mixed with 7.5 mL of SGF (stimulated gastric fluid), 1.6 mL of pepsin solution (25000 U/mL, Sigma-Aldrich) prepared in the SGF solution, 5 mL of 0.3 M CaCl_2_ and 0.695 mL of water. pH was adjusted to 3.0 by adding 1M HCl. The mixture was left at 37 °C for 2 h in a shaking incubator (300 rpm). Intestinal phase: 11 mL of SIF (stimulated intestinal fluid), 5 mL of pancreatic solution prepared in SIF (800 U/mL, Sigma-Aldrich, Saint Louis, MO, USA), 2.5 mL of freshly prepared bile solution (160 mM, Sigma), 40 µL of 0.3 M CaCl_2_ were added and the pH was adjusted to 7.0 by adding 1 M NaOH. The mixture was made up to a final volume of 40 mL with water. The samples were incubated in a shaking incubator (300 rpm) at 37 °C for an additional 2 h. After completion of digestion, the samples were centrifuged at 4500× *g* for 10 min at 4 °C, and the supernatant was collected and stored at −80 °C until the antiproliferation experiment. The composition of SSF, SGF, and SIF is shown in Table S1 (Supplementary Material).

### 3.6. Statistical Analysis

Experiments were performed in triplicate. All results were presented as mean ± SD. Statistical analysis was performed using GraphPad Prism Version 9. The mean values were compared using two-way ANOVA, followed by Tukey’s and Dunnett’s multiple comparison test for antiproliferative activity of waste extracts and HPLC analysis and Šídák’s multiple comparison test for antiproliferative activity of quercetin conjugates and antioxidant analysis. Differences were considered significant at * p < 0.05, ** p < 0.01, *** p < 0.001 and **** p < 0.0001.

## 4. Conclusions

This was the first study of the chemical composition and biological activity of peel extracts from triploid onion *Allium × cornutum* (Ljutika). The results showed that the peel extracts of traditionally grown Dalmatian onions, *A. × cornutum* and two varieties of *A. cepa,* are rich in flavonols (the main antioxidant compounds) and possess antioxidant, antimicrobial and antiproliferative properties. Considering that a large amount of onion peel waste is generated annually, which is an increasing environmental and economic problem, new studies, including the present one, should provide insight into the possibility of their use as a source of biologically active phytochemicals that can be exploited in the pharmaceutical, cosmetic and food industries. However, further research is needed to identify other biologically active compounds present in onion wastes and the mechanism behind their biological activity.

## Figures and Tables

**Table 1:** HPLC quantification of flavonols and anthocyanins in two varieties of *A. cepa* and *A. × cornutum* extracts.

	*A. × cornutum* (t_R_, min)	*A. cepa* (Yellow Variety) (t_R_, min)	*A. cepa* (Red Variety) (t_R_, min)
**Flavonols ^a^**			
Quercetin 3,4′-diglucoside (1)	618.75 ± 0.36 ^a^ (32.52)	100.40 ± 0.05 ^c^ (32.46)	331.93 ± 0.12 ^b^ (32.50)
Quercetin 4′-monoglucoside (2)	617.01 ± 0.40 ^a^ (40.47)	140.43 ± 0.10 ^c^ (40.42)	298.87 ± 0.13 ^b^ (40.46)
Myricetin (3)	14.88 ± 0.01 ^a^ (42.35)	8.63 ± 0.01 ^c^ (42.31)	9.31 ± 0.02 ^b^ (42.35)
Quercetin aglycone (4)	297.21 ± 0.40 ^a^ (47.14)	60.51 ± 0.06 ^c^ (47.09)	70.10 ± 0.08 ^b^ (47.14)
Isorhamnetin (5)	32.07 ± 0.04 ^a^ (52.29)	2.21 ± 0.01 ^c^ (52.23)	13.73 ± 0.01 ^b^ (52.28)
**Anthocyanins ^a^**			
Peonidin 3′-glucoside	Nd	1.11 ± 0.00 (12.38)	Nd
Peonidin 3′-glucoside acetate	Nd	Nd	0.67 ± 0.3 (25.02)
Delphinidin 3′-glucoside acetate	0.23 ± 0.00 (18.71)	Nd	Nd
Malvidin 3′-glucoside	0.05 ± 0.00 ^b^ (13.44)	0.53 ± 0.00 ^a^ (12.84)	0.24 ± 0.00 ^b^ (13.28)
Cyanidin 3′-glucoside	0.32 ± 0.01 ^b^ (8.47)	7.85 ± 0.11 ^a^ (8.56)	0.11 ± 0.00 ^b^ (8.06)
Cyanidin 3′-glucoside acetate	1.22 ± 0.01 ^b^ (21.81)	0.76 ± 0.00 ^c^ (22.43)	3.44 ± 0.03 ^a^ (21.75)
Petunidin 3′-glucoside	Nd	0.12 ± 0.00 (10.58)	Nd
Petunidin 3′-glucoside acetate	Nd	Nd	0.17 ± 0.02 (23.25)

^a^ Concentration in mg/100 g dry weight; t_R_, retention time; Nd, not determined. Results are means ± SD (n = 3). Different superscript letters (a,b,c) indicate a statistically significant difference between three onion waste extracts (*p* < 0.05) (Data S1).

**Table 2:** Antioxidant potential of *A. **×** cornutum* and two varieties of *A. cepa* L. peel extracts determined by ORAC and DPPH method.

Antioxidant Assay	*A. × cornutum*	*A. cepa* (Yellow Variety)		*A. cepa* (Red Variety)
ORAC (Trolox eq)	20.5 ± 0.17 ^a^	12.98 ± 0.29 ^b^	4.64 ± 0.34 ^c^	
DPPH (% inhibition)	82.18 ± 1.09 ^a^	65.76 ± 2.97 ^b^	53.43 ± 4.36 ^c^	

Results are means ± SD (n = 3). Different superscript letters (a,b,c) indicate a statistically significant difference between three onion waste extracts (*p* < 0.05) (Data S2).

**Table 3:** Antimicrobial activity of *A. × cornutum* and *A. cepa* (yellow and red variety) waste extracts.

Species	Strain Origin	*Allium × cornutum* ^a^		*Allium cepa* (Yellow Variety) ^a^		*Allium cepa* (Red Variety) ^a^	
**Gram-Positive bacteria**		MIC	MBC	MIC	MBC	MIC	MBC
*Staphylococcus aureus*	ATCC 29213	7.8	125	7.8	125	500	500
*Staphylococcus aureus*	Clinical/MRSA	31.25	125	62.5	125	500	500
*Enterococcus faecalis*	ATCC 29212	250	250	250	500	1000	2000
*Streptococcus pyogenes*	ATCC 19615	31.25	125	125	250	500	500
*Listeria monocytogenes*	ATCC 19111 (1/2a)	15.6	125	250	500	1000	1000
*Bacillus cereus*	Food	125	125	250	500	500	500
**Gram-Negative bacteria**							
*Escherichia coli*	ATCC 25922	500	500	500	2000	>2000	>2000
*Klebsiella pneumoniae*	ATCC 13883	500	2000	1000	>2000	>2000	>2000
**Yeast**		MIC_50_	MIC_90_	MIC_50_	MIC_90_	MIC_50_	MIC_90_
*Candida albicans*	ATCC 90029	10000	2000	>2000	>2000	>2000	>2000
**Mould**							
*Aspergillus niger*	Food	500	2000	1000	>2000	1000	>2000

^a^ Extracts of *A. × cornutum* and *A. cepa* (yellow and red variety) (c = 10 mg/mL) were tested in the final concentration range from 2000 to 1.95 µg/mL. MIC—minimum inhibitory concentration; MBC—minimum bactericidal concentration.

**Table 4:** Antimicrobial activity of quercetin 3,4′-diglucoside and quercetin 4′-monoglucoside.

Species	Strain Origin	Quercetin 4′-Monglucoside ^b^		Quercetin 3,4′-Diglucoside ^b^	
**Gram-Positive Bacteria**		MIC	MBC	MIC	MBC
*Staphylococcus aureus*	ATCC 29213	>300	>300	>200	>200
*Staphylococcus aureus*	Clinical/MRSA	>300	>300	>200	>200
*Enterococcus faecalis*	ATCC 29212	150	>300	200	>200
*Streptococcus pyogenes*	ATCC 19615	>300	>300	>200	>200
*Listeria monocytogenes*	ATCC 19111 (1/2a)	>300	>300	>200	>200
*Bacillus cereus*	Food	>300	>300	>200	>200
**Gram-Negative Bacteria**					
*Escherichia coli*	ATCC 25922	>300	>300	>200	>200
*Klebsiella pneumoniae*	ATCC 13883	>300	>300	>200	>200
**Yeast**		MIC_50_	MIC_90_	MIC_50_	MIC_90_
*Candida albicans*	ATCC 90029	300	>300	>200	>200
**Mould**					
*Aspergillus niger*	Food	150	>300	200	>200

^b^ Quercetin 4′-monoglucoside (c = 1.5 mg/mL) was tested in the concentration range of 300 to 0.3 µg/mL, while in the case of quercetin 3,4′-diglucoside (c = 1 mg/mL) the concentration range was 200 to 1.9 µg/mL. MIC—minimum inhibitory concentration, MBC—minimum bactericidal concentration.

## Data Availability

All data is confirmed as original.

## Supplementary Materials

The following are available online at https://www.mdpi.com/article/10.3390/plants10050832/s1, Figure S1: High-performance liquid chromatography (HPLC) of methanolic waste extracts of *A. cepa* red variety, Data S1: Statistical analysis of HPLC quantification of flavonols and anthocyanins, Data S2: Statistical analysis of antioxidant activity, Data S3: Statistical analysis of antiproliferative activity of onion waste extracts, Data S4: Statistical analysis of antiproliferative activity of quercetin conjugates, Data S5: Statistical analysis of antiproliferative activity of *A. × cornutum* after digestion, Data S6: Statistical analysis of antiproliferative activity of *A. cepa* (yellow variety) after digestion, Data S7: Statistical analysis of antiproliferative activity of *A. cepa* (red variety) after digestion, Data S8-1: Quality parameters of the optimized HPLC for flavonol quantification, Data S8-2: Stability data detection and quantification limits of 5 flavonoid standards analyzed by HPLC, Data S8-3: Stability data detection and quantification limits of four anthocyanins standard analysed by HPLC, Data S8-4: Quality parameters of the optimized HPLC for anthocyanin quantification, Table S1: Preparation of stock solutions of stimulated digestive fluids.
