# Supplementary material for: Phytochemical Characterization and Screening of Antioxidant, Antimicrobial and Antiproliferative Properties of Allium × cornutum Clementi and Two Varieties of Allium cepa L. Peel Extracts
Source: Plants (Basel). 2021 Apr 21;10(5):832. doi: 10.3390/plants10050832 (PMC8143352; doi:10.3390/plants10050832)
Supplement: Supplementary file 1 [file plants-10-00832-s001.zip › plants-1161531-supplementary.pdf]

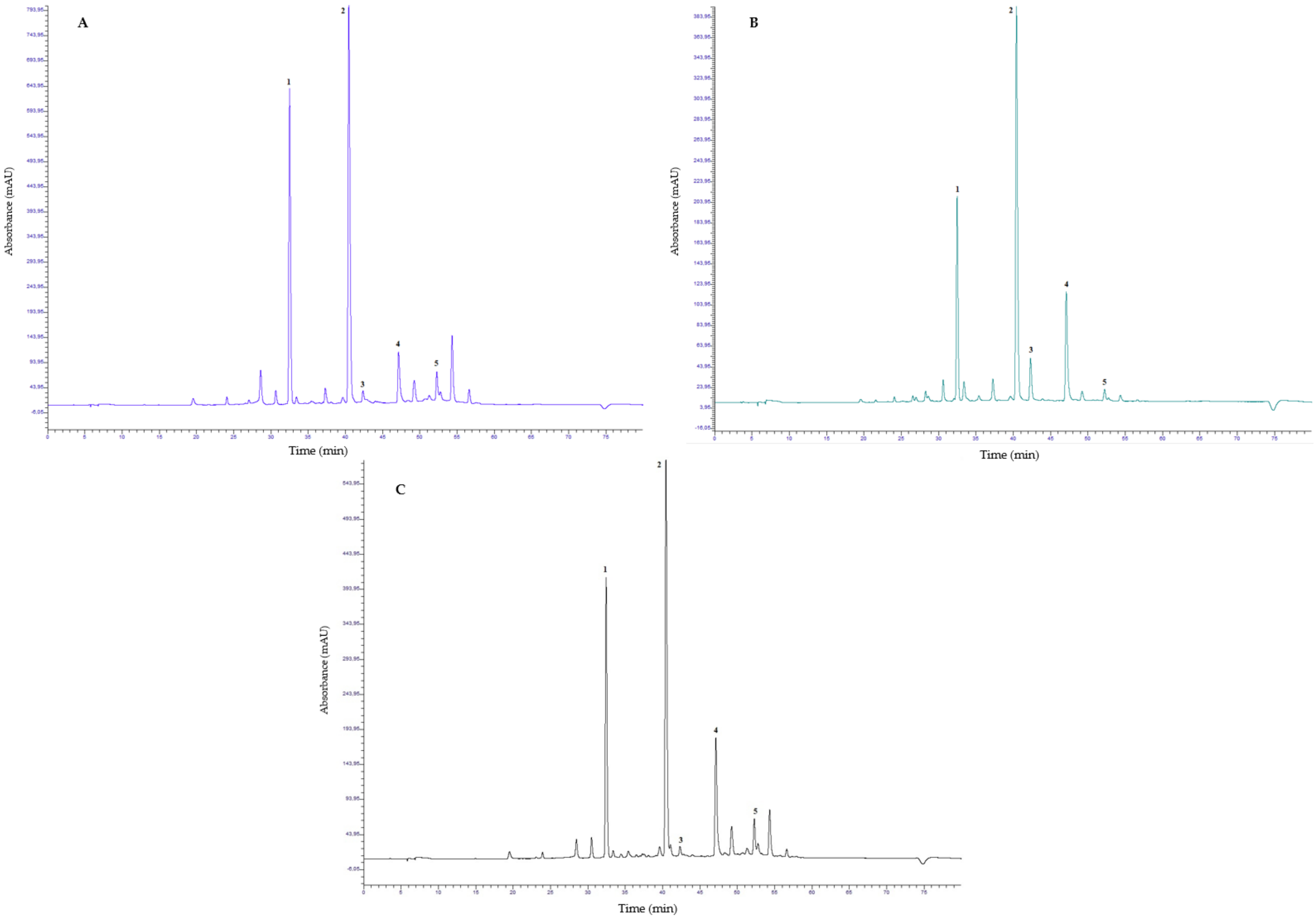

Figure S1. High-performance liquid chromatography (HPLC) of methanolic waste extracts of *A. cepa* red variety (**A**), *A. cepa* yellow variety (**B**) and *A.* × *cornutum* (**C**). Peaks shown are: (**1**) quercetin 3,4'-diglucoside; (**2**) quercetin 4'-monoglucoside; (**3**) myricetin, (**4**) quercetin aglycone and (**5**) isorhamnetin. *A.* × *cornutum* extract was diluted three times.

Data S1_Statistical analysis of HPLC quantification of flavonols and anthocyanins

Within each row, compare columns (simple effects within rows)

Number of families 13
Number of comparisons per family 3
Alpha 0,05

Tukey's multiple comparisons test Mean Diff, 95,00% CI of diff, Significant? Summary Adjusted P Value

Quercetin 3,4′-diglucoside
A. × cornutum vs. A. cepa (yellow variety) 518,4 518,1 to 518,6 Yes **** <0,0001

A. × cornutum vs. A. cepa (red variety) 286,8 286,6 to 287,1 Yes **** <0,0001
A. cepa (yellow variety) vs. A. cepa (red variety) -231,5 -231,8 to -231,3 Yes **** <0,0001

Quercetin 4′-monoglucoside
A. × cornutum vs. A. cepa (yellow variety) 476,6 476,3 to 476,8 Yes **** <0,0001

A. × cornutum vs. A. cepa (red variety) 318,1 317,9 to 318,4 Yes **** <0,0001
A. cepa (yellow variety) vs. A. cepa (red variety) -158,4 -158,7 to -158,2 Yes **** <0,0001

Myricetin
A. × cornutum vs. A. cepa (yellow variety) 6,250 6,007 to 6,493 Yes **** <0,0001

A. × cornutum vs. A. cepa (red variety) 5,570 5,327 to 5,813 Yes **** <0,0001
A. cepa (yellow variety) vs. A. cepa (red variety) -0,6800 -0,9226 to -0,4374 Yes **** <0,0001

Quercetin aglycone
A. × cornutum vs. A. cepa (yellow variety) 236,7 236,5 to 236,9 Yes **** <0,0001

A. × cornutum vs. A. cepa (red variety) 227,1 226,9 to 227,4 Yes **** <0,0001
A. cepa (yellow variety) vs. A. cepa (red variety) -9,590 -9,833 to -9,347 Yes **** <0,0001

Isorhamnetin

A. × cornutum vs. A. cepa (yellow variety)	29,86	29,62 to 30,10	Yes	****	<0,0001
A. × cornutum vs. A. cepa (red variety)	18,34	18,10 to 18,58	Yes	****	<0,0001
A. cepa (yellow variety) vs. A. cepa (red variety)	-11,52	-11,76 to -11,28	Yes	****	<0,0001

Peonidin 3′-glucoside

A. × cornutum vs. A. cepa (yellow variety)	-1,110	-1,353 to -0,8674	Yes	****	<0,0001
A. × cornutum vs. A. cepa (red variety)	-4,263e-014	-0,2426 to 0,2426	No	ns	>0,9999
A. cepa (yellow variety) vs. A. cepa (red variety)	1,110	0,8674 to 1,353	Yes	****	<0,0001

Peonidin 3′-glucoside acetate

A. × cornutum vs. A. cepa (yellow variety)	0,000	-0,2426 to 0,2426	No	ns	>0,9999
A. × cornutum vs. A. cepa (red variety)	-0,6700	-0,9126 to -0,4274	Yes	****	<0,0001
A. cepa (yellow variety) vs. A. cepa (red variety)	-0,6700	-0,9126 to -0,4274	Yes	****	<0,0001

Delphinidin 3′-glucoside acetate

A. × cornutum vs. A. cepa (yellow variety)	0,2300	-0,01257 to 0,4726	No	ns	0,0668
A. × cornutum vs. A. cepa (red variety)	0,2300	-0,01257 to 0,4726	No	ns	0,0668
A. cepa (yellow variety) vs. A. cepa (red variety)	0,000	-0,2426 to 0,2426	No	ns	>0,9999

Malvidin 3′-glucoside

A. × cornutum vs. A. cepa (yellow variety)	-0,4800	-0,7226 to -0,2374	Yes	****	<0,0001
A. × cornutum vs. A. cepa (red variety)	-0,1900	-0,4326 to 0,05257	No	ns	0,1538

A. cepa (yellow variety) vs. A. cepa (red variety) 0,2900 0,04743 to 0,5326 Yes * 0,0150

Cyanidin 3′-glucoside

A. × cornutum vs. A. cepa (yellow variety) -7,530 -7,773 to -7,287 Yes **** <0,0001

A. × cornutum vs. A. cepa (red variety) 0,2100 -0,03257 to 0,4526 No ns 0,1031

A. cepa (yellow variety) vs. A. cepa (red variety) 7,740 7,497 to 7,983 Yes **** <0,0001

Cyanidin 3′-glucoside acetate

A. × cornutum vs. A. cepa (yellow variety) 0,4600 0,2174 to 0,7026 Yes **** <0,0001

A. × cornutum vs. A. cepa (red variety) -2,220 -2,463 to -1,977 Yes **** <0,0001

A. cepa (yellow variety) vs. A. cepa (red variety) -2,680 -2,923 to -2,437 Yes **** <0,0001

Petunidin 3′-glucoside

A. × cornutum vs. A. cepa (yellow variety) -0,1200 -0,3626 to 0,1226 No ns 0,4674

A. × cornutum vs. A. cepa (red variety) 0,000 -0,2426 to 0,2426 No ns >0,9999

A. cepa (yellow variety) vs. A. cepa (red variety) 0,1200 -0,1226 to 0,3626 No ns 0,4674

Petunidin 3′-glucoside acetate

A. × cornutum vs. A. cepa (yellow variety) 0,000 -0,2426 to 0,2426 No ns >0,9999

A. × cornutum vs. A. cepa (red variety) -0,1700 -0,4126 to 0,07257 No ns 0,2215

A. cepa (yellow variety) vs. A. cepa (red variety) -0,1700 -0,4126 to 0,07257 No ns 0,2215

Test details Mean 1 Mean 2 Mean Diff, SE of diff, N1 N2 q DF

Quercetin 3,4′-diglucoside

A. × cornutum vs. A. cepa (yellow variety)	618,8	100,4	518,4	0,1015	1	1	7220	78,00
A. × cornutum vs. A. cepa (red variety)	618,8	331,9	286,8	0,1015	1	1	3995	78,00
A. cepa (yellow variety) vs. A. cepa (red variety)	100,4	331,9	-231,5	0,1015	1	1	3225	78,00

Quercetin 4′-monoglucoside

A. × cornutum vs. A. cepa (yellow variety)	617,0	140,4	476,6	0,1015	1	1	6639	78,00
A. × cornutum vs. A. cepa (red variety)	617,0	298,9	318,1	0,1015	1	1	4432	78,00
A. cepa (yellow variety) vs. A. cepa (red variety)	140,4	298,9	-158,4	0,1015	1	1	2207	78,00

Myricetin

A. × cornutum vs. A. cepa (yellow variety)	14,88	8,630	6,250	0,1015	1	1	87,06	78,00
A. × cornutum vs. A. cepa (red variety)	14,88	9,310	5,570	0,1015	1	1	77,59	78,00
A. cepa (yellow variety) vs. A. cepa (red variety)	8,630	9,310	-0,6800	0,1015	1	1	9,472	78,00

Quercetin aglycone

A. × cornutum vs. A. cepa (yellow variety)	297,2	60,51	236,7	0,1015	1	1	3297	78,00
A. × cornutum vs. A. cepa (red variety)	297,2	70,10	227,1	0,1015	1	1	3164	78,00
A. cepa (yellow variety) vs. A. cepa (red variety)	60,51	70,10	-9,590	0,1015	1	1	133,6	78,00

Isorhamnetin

A. × cornutum vs. A. cepa (yellow variety)	32,07	2,210	29,86	0,1015	1	1	415,9	78,00
A. × cornutum vs. A. cepa (red variety)	32,07	13,73	18,34	0,1015	1	1	255,5	78,00
A. cepa (yellow variety) vs. A. cepa (red variety)	2,210	13,73	-11,52	0,1015	1	1	160,5	78,00

Peonidin 3′-glucoside

A. × cornutum vs. A. cepa (yellow variety)	-4,263e-014	1,110	-1,110	0,1015	1	1	15,46	78,00
A. × cornutum vs. A. cepa (red variety)	-4,263e-014	0,000	-4,263e-014	0,1015	1	1	5,938e-013	78,00
A. cepa (yellow variety) vs. A. cepa (red variety)	1,110	0,000	1,110	0,1015	1	1	15,46	78,00

Peonidin 3′-glucoside acetate

A. × cornutum vs. A. cepa (yellow variety)	0,000	0,000	0,000	0,1015	1	1	0,000	78,00
A. × cornutum vs. A. cepa (red variety)	0,000	0,6700	-0,6700	0,1015	1	1	9,333	78,00
A. cepa (yellow variety) vs. A. cepa (red variety)	0,000	0,6700	-0,6700	0,1015	1	1	9,333	78,00
Delphinidin 3′-glucoside acetate								
A. × cornutum vs. A. cepa (yellow variety)	0,2300	0,000	0,2300	0,1015	1	1	3,204	78,00
A. × cornutum vs. A. cepa (red variety)	0,2300	0,000	0,2300	0,1015	1	1	3,204	78,00
A. cepa (yellow variety) vs. A. cepa (red variety)	0,000	0,000	0,000	0,1015	1	1	0,000	78,00
Malvidin 3′-glucoside								
A. × cornutum vs. A. cepa (yellow variety)	0,05000	0,5300	-0,4800	0,1015	1	1	6,686	78,00
A. × cornutum vs. A. cepa (red variety)	0,05000	0,2400	-0,1900	0,1015	1	1	2,647	78,00
A. cepa (yellow variety) vs. A. cepa (red variety)	0,5300	0,2400	0,2900	0,1015	1	1	4,040	78,00
Cyanidin 3′-glucoside								
A. × cornutum vs. A. cepa (yellow variety)	0,3200	7,850	-7,530	0,1015	1	1	104,9	78,00
A. × cornutum vs. A. cepa (red variety)	0,3200	0,1100	0,2100	0,1015	1	1	2,925	78,00
A. cepa (yellow variety) vs. A. cepa (red variety)	7,850	0,1100	7,740	0,1015	1	1	107,8	78,00
Cyanidin 3′-glucoside acetate								
A. × cornutum vs. A. cepa (yellow variety)	1,220	0,7600	0,4600	0,1015	1	1	6,408	78,00
A. × cornutum vs. A. cepa (red variety)	1,220	3,440	-2,220	0,1015	1	1	30,92	78,00
A. cepa (yellow variety) vs. A. cepa (red variety)	0,7600	3,440	-2,680	0,1015	1	1	37,33	78,00
Petunidin 3′-glucoside								

A. × cornutum vs. A. cepa (yellow variety)	0,000	0,1200	-0,1200	0,1015	1	1	1,672	78,00
A. × cornutum vs. A. cepa (red variety)	0,000	0,000	0,000	0,1015	1	1	0,000	78,00
A. cepa (yellow variety) vs. A. cepa (red variety)	0,1200	0,000	0,1200	0,1015	1	1	1,672	78,00
Petunidin 3′-glucoside acetate								
A. × cornutum vs. A. cepa (yellow variety)	0,000	0,000	0,000	0,1015	1	1	0,000	78,00
A. × cornutum vs. A. cepa (red variety)	0,000	0,1700	-0,1700	0,1015	1	1	2,368	78,00
A. cepa (yellow variety) vs. A. cepa (red variety)	0,000	0,1700	-0,1700	0,1015	1	1	2,368	78,00

Data S2_Statistical analysis of antioxidant activity

Within each row, compare columns (simple effects within rows)

Number of families 2
Number of comparisons per family 3
Alpha 0,05

Šídák's multiple comparisons test	Mean Diff,	95,00% CI of diff,	Below threshold?	Summary	Adjusted P Value
ORAC (Trolox eq)					
A. × cornutum vs. A. cepa (yellow variety)	7,520	2,526 to 12,51	Yes	**	0,0039
A. × cornutum vs. A. cepa (red variety)	15,86	10,87 to 20,85	Yes	****	<0,0001
A. cepa (yellow variety) vs. A. cepa (red variety)	8,340	3,346 to 13,33	Yes	**	0,0018
DPPH (% inhibition)					
A. × cornutum vs. A. cepa (yellow variety)	16,42	11,43 to 21,41	Yes	****	<0,0001
A. × cornutum vs. A. cepa (red variety)	28,75	23,76 to 33,74	Yes	****	<0,0001
A. cepa (yellow variety) vs. A. cepa (red variety)	12,33	7,336 to 17,32	Yes	****	<0,0001

Test details	Mean 1	Mean 2	Mean Diff,	SE of diff,	N1	N2	t	DF
ORAC (Trolox eq)								
A. × cornutum vs. A. cepa (yellow variety)	20,50	12,98	7,520	1,803	3	3	4,172	12,00
A. × cornutum vs. A. cepa (red variety)	20,50	4,640	15,86	1,803	3	3	8,798	12,00
A. cepa (yellow variety) vs. A. cepa (red variety)	12,98	4,640	8,340	1,803	3	3	4,626	12,00
DPPH (% inhibition)								
A. × cornutum vs. A. cepa (yellow variety)	82,18	65,76	16,42	1,803	3	3	9,109	12,00
A. × cornutum vs. A. cepa (red variety)	82,18	53,43	28,75	1,803	3	3	15,95	12,00
A. cepa (yellow variety) vs. A. cepa (red variety)	65,76	53,43	12,33	1,803	3	3	6,840	12,00

Data S3_Statistical analysis of antiproliferative activity of onion waste extracts

Within each row, compare columns (simple effects within rows)

Number of families 3
Number of comparisons per family 3
Alpha 0,05

Tukey's multiple comparisons test	Mean Diff,	95,00% CI of diff,	Below threshold?	Summary	Adjusted P Value
HeLa					
A. × cornutum vs. A. cepa (red variety)	-3,435	-11,52 to 4,648	No	ns	0,4897
A. × cornutum vs. A. cepa (yellow variety)	-2,765	-10,85 to 5,318	No	ns	0,6214
A. cepa (red variety) vs. A. cepa (yellow variety)	0,6700	-7,413 to 8,753	No	ns	0,9710
HCT116					
A. × cornutum vs. A. cepa (red variety)	0,4550	-7,628 to 8,538	No	ns	0,9865
A. × cornutum vs. A. cepa (yellow variety)	-5,880	-13,96 to 2,203	No	ns	0,1603
A. cepa (red variety) vs. A. cepa (yellow variety)	-6,335	-14,42 to 1,748	No	ns	0,1268
U2OS					
A. × cornutum vs. A. cepa (red variety)	0,4197	-7,664 to 8,503	No	ns	0,9885
A. × cornutum vs. A. cepa (yellow variety)	-1,353	-9,436 to 6,731	No	ns	0,8881
A. cepa (red variety) vs. A. cepa (yellow variety)	-1,773	-9,856 to 6,311	No	ns	0,8172

Test details	Mean 1	Mean 2	Mean Diff,	SE of diff,	N1	N2	q	DF

HeLa								
A. × cornutum vs. A. cepa (red variety)	21,04	24,48	-3,435	2,895	2	2	1,678	9,000
A. × cornutum vs. A. cepa (yellow variety)	21,04	23,81	-2,765	2,895	2	2	1,351	9,000
A. cepa (red variety) vs. A. cepa (yellow variety)	24,48	23,81	0,6700	2,895	2	2	0,3273	9,000
HCT116								
A. × cornutum vs. A. cepa (red variety)	30,40	29,94	0,4550	2,895	2	2	0,2223	9,000
A. × cornutum vs. A. cepa (yellow variety)	30,40	36,28	-5,880	2,895	2	2	2,872	9,000
A. cepa (red variety) vs. A. cepa (yellow variety)	29,94	36,28	-6,335	2,895	2	2	3,094	9,000
U2OS								
A. × cornutum vs. A. cepa (red variety)	25,40	24,98	0,4197	2,895	2	2	0,2050	9,000
A. × cornutum vs. A. cepa (yellow variety)	25,40	26,75	-1,353	2,895	2	2	0,6608	9,000
A. cepa (red variety) vs. A. cepa (yellow variety)	24,98	26,75	-1,773	2,895	2	2	0,8658	9,000

Data S4_Statistical analysis of antiproliferative activity of quercetin conjugates

Compare each cell mean with the other cell mean in that row

Number of families 1
Number of comparisons per family 3
Alpha 0,05

Šídák's multiple comparisons test	Mean Diff,	95,00% CI of diff,	Below threshold?	Summary	Adjusted P Value
quercetin 4'-monoglucoside - quercetin 3,4'-diglucoside					
HeLa	-225,2	-229,6 to -220,8	Yes	****	<0,0001
HCT116	-13,07	-17,44 to -8,690	Yes	***	0,0002
U2OS	-50,60	-54,97 to -46,22	Yes	****	<0,0001

Test details	Mean 1	Mean 2	Mean Diff,	SE of diff,	N1	N2	t	DF
quercetin 4'-monoglucoside - quercetin 3,4'-diglucoside								
HeLa	95,12	320,3	-225,2	1,336	2	2	168,5	6,000
HCT116	104,2	117,3	-13,07	1,336	2	2	9,778	6,000
U2OS	106,9	157,4	-50,60	1,336	2	2	37,86	6,000

Data S5_Statistical analysis of antiproliferative activity of A. x cornutum after digestion

Within each row, compare columns (simple effects within rows)

Number of families 3
Number of comparisons per family 3
Alpha 0,05

Dunnett's multiple comparisons test	Mean Diff,	95,00% CI of diff,	Below threshold?	Summary	Adjusted P Value
Hela					
Undigested vs. OF	-1,335	-5,980 to 3,310	No	ns	0,7840
Undigested vs. GF	-0,2750	-4,920 to 4,370	No	ns	0,9971
Undigested vs. IF	5,850	1,205 to 10,50	Yes	*	0,0143
HCT116					
Undigested vs. OF	0,4300	-4,215 to 5,075	No	ns	0,9890
Undigested vs. GF	-3,050	-7,695 to 1,595	No	ns	0,2352
Undigested vs. IF	5,545	0,8996 to 10,19	Yes	*	0,0197
U2OS					
Undigested vs. OF	0,8350	-3,810 to 5,480	No	ns	0,9303
Undigested vs. GF	0,5400	-4,105 to 5,185	No	ns	0,9789
Undigested vs. IF	5,185	0,5396 to 9,830	Yes	*	0,0286

Test details	Mean 1	Mean 2	Mean Diff,	SE of diff,	N1	N2	q	DF
Hela								
Undigested vs. OF	23,77	25,10	-1,335	1,731	2	2	0,7710	12,00
Undigested vs. GF	23,77	24,04	-0,2750	1,731	2	2	0,1588	12,00
Undigested vs. IF	23,77	17,92	5,850	1,731	2	2	3,379	12,00
HCT116								
Undigested vs. OF	32,77	32,34	0,4300	1,731	2	2	0,2483	12,00
Undigested vs. GF	32,77	35,82	-3,050	1,731	2	2	1,762	12,00
Undigested vs. IF	32,77	27,23	5,545	1,731	2	2	3,202	12,00

U2OS								
Undigested vs. OF	33,14	32,31	0,8350	1,731	2	2	0,4822	12,00
Undigested vs. GF	33,14	32,60	0,5400	1,731	2	2	0,3119	12,00
Undigested vs. IF	33,14	27,96	5,185	1,731	2	2	2,995	12,00

Data S6_Statistical analysis of antiproliferative activity of A. cepa (yellow variety) after digestion

Within each row, compare columns (simple effects within rows)

Number of families 3
Number of comparisons per family 3
Alpha 0,05

Dunnett's multiple comparisons test	Mean Diff,	95,00% CI of diff,	Below threshold?	Summary	Adjusted P Value
Hela					
Undigested vs. OF	-0,1950	-4,209 to 3,819	No	ns	0,9983
Undigested vs. GF	0,6550	-3,359 to 4,669	No	ns	0,9460
Undigested vs. IF	6,610	2,596 to 10,62	Yes	**	0,0023
HCT116					
Undigested vs. OF	-0,4600	-4,474 to 3,554	No	ns	0,9797
Undigested vs. GF	-2,455	-6,469 to 1,559	No	ns	0,2819
Undigested vs. IF	8,670	4,656 to 12,68	Yes	***	0,0003
U2OS					
Undigested vs. OF	0,02000	-3,994 to 4,034	No	ns	>0,9999
Undigested vs. GF	-1,200	-5,214 to 2,814	No	ns	0,7648
Undigested vs. IF	4,455	0,4414 to 8,469	Yes	*	0,0295

Test details	Mean 1	Mean 2	Mean Diff,	SE of diff,	N1	N2	q	DF
Hela								
Undigested vs. OF	25,00	25,19	-0,1950	1,496	2	2	0,1303	12,00
Undigested vs. GF	25,00	24,34	0,6550	1,496	2	2	0,4378	12,00
Undigested vs. IF	25,00	18,39	6,610	1,496	2	2	4,418	12,00
HCT116								
Undigested vs. OF	37,92	38,38	-0,4600	1,496	2	2	0,3075	12,00
Undigested vs. GF	37,92	40,37	-2,455	1,496	2	2	1,641	12,00
Undigested vs. IF	37,92	29,25	8,670	1,496	2	2	5,795	12,00

U2OS

Undigested vs. OF	36,91	36,89	0,02000	1,496	2	2	0,01337	12,00
Undigested vs. GF	36,91	38,11	-1,200	1,496	2	2	0,8021	12,00
Undigested vs. IF	36,91	32,45	4,455	1,496	2	2	2,978	12,00

Data S7_Statistical analysis of antiproliferative activity of A. cepa (red variety) after digestion

Within each row, compare columns (simple effects within rows)

Number of families 3
Number of comparisons per family 3
Alpha 0,05

Dunnett's multiple comparisons test	Mean Diff,	95,00% CI of diff,	Below threshold?	Summary	Adjusted P Value
Hela					
Undigested vs. OF	-1,145	-4,251 to 1,961	No	ns	0,6449
Undigested vs. GF	-1,075	-4,181 to 2,031	No	ns	0,6842
Undigested vs. IF	6,960	3,854 to 10,07	Yes	***	0,0002
HCT116					
Undigested vs. OF	0,01000	-3,096 to 3,116	No	ns	>0,9999
Undigested vs. GF	-2,025	-5,131 to 1,081	No	ns	0,2396
Undigested vs. IF	4,275	1,169 to 7,381	Yes	**	0,0081
U2OS					
Undigested vs. OF	0,1150	-2,991 to 3,221	No	ns	0,9993
Undigested vs. GF	0,5950	-2,511 to 3,701	No	ns	0,9179
Undigested vs. IF	5,675	2,569 to 8,781	Yes	***	0,0010

Test details	Mean 1	Mean 2	Mean Diff,	SE of diff,	N1	N2	q	DF
Hela								
Undigested vs. OF	26,11	27,25	-1,145	1,158	2	2	0,9891	12,00
Undigested vs. GF	26,11	27,18	-1,075	1,158	2	2	0,9287	12,00
Undigested vs. IF	26,11	19,15	6,960	1,158	2	2	6,013	12,00
HCT116								
Undigested vs. OF	29,92	29,91	0,01000	1,158	2	2	0,008639	12,00
Undigested vs. GF	29,92	31,94	-2,025	1,158	2	2	1,749	12,00
Undigested vs. IF	29,92	25,64	4,275	1,158	2	2	3,693	12,00

U2OS

Undigested vs. OF	32,42	32,30	0,1150	1,158	2	2	0,09935	12,00
Undigested vs. GF	32,42	31,82	0,5950	1,158	2	2	0,5140	12,00
Undigested vs. IF	32,42	26,74	5,675	1,158	2	2	4,903	12,00

Data S8-1: Quality parameters of the optimized HPLC for flavonol quantification (n = 6).

**Flavonols**	**Quercetin calibration range (mg/L)**	**r^2**
(1) Quercetin 3.4-diglucoside	2.48-158.40	0.999981
(2) Quercetin 4-monoglucoside	1.88-120	0.999990
(3) Quercetin	2.11-135.1	0.999841
(4) Isorhamnetin	0.66-42.46	0.999967
(5) Myricetin	0.58-36.8	0.999968
(6) Kaempferol	0.41-26.28	0.999926

Data S8-2: Stability data. detection and quantification limits of 5 flavonoid standards analyzed by HPLC

**Component name**	**Time [min]**	**Area [Uv*sec]**	**Area [%]**	**LOD (µg/ml)**	**LOQ (µg/mL)**
(1) quercetin 3.4-diglucoside	33.322	19564.66	24.33	0.133	0.339
(2) quercetin 4-monoglucoside	41.471	23154.07	28.79	0.07	0.21
(3) quercetin	48.185	24298.08	30.22	0.038	0.114
(4) isorhamnetin	52.933	4323.8	5.38	0.081	0.243
(5) kaempferol	52.24	9072.13	11.28	0.032	0.096

Data S8-3: Stability data. detection and quantification limits of four anthocyanins standard analysed by HPLC

**Anthocyanins**	**Retention time (min)**	**Area (counts)**	**Result**	**LOD (µg/ml)**	**LOQ (µg/ml)**
(1) Malvidin 3-glucoside	13.808	10731967	22.4944	0.032	0.066
(2) Cyanidin 3-glucoside	8.605	2222866	164.8706		
(3) Petunidin 3-glucoside	10.072	2789539	684.6994		
(4) Delphinidin 3-glucoside	19.907	98032	70.8784		

Data S8-4: Quality parameters of the optimized HPLC for anthocyanin quantification (n = 1)

**Flavonols**	**Malvidin 3-glucoside calibration range (mg/L)**	**r^2**
(1) Malvidin 3-glucoside	0.208-100	0.9993

Table S1. Preparation of stock solutions of stimulated digestive fluids.

Compound	Stock concentration		SSF pH 7.0 Concentration in SSF	SGF pH 3.0 Concentration in SGF	SIF pH 7.0 Concentration in SIF
	g/l	mol/l	mmol/l	mmol/l	mmol/l
KCl	37.3	0.5	15.1	6.9	6.8
KH_2PO_4	68	0.5	3.7	0.9	0.8
$NaHCO_3$	84	1	13.6	25	85
NaCl	117	2	-	47.2	38.4
$MgCl_2(H_2O)_6$	30.5	0.15	0.15	0.1	0.33
$(NH_4)_2CO_3$	48	0.5	0.06	0.5	-
NaOH	-	1	-	-	8.4
HCl	-	6	1.1	15.6	-
$CaCl_2(H_2O)_2$	44.1	0.3	(0.75)	(0.075)	(0.3)

SSF- stimulated salivary fluid; SGF- stimulated gastric fluid; SIF- stimulated intestinal fluid
